# Understanding providers’ attitudes and key concerns toward incorporating CVD risk prediction into clinical practice: a qualitative study

**DOI:** 10.1186/s12913-021-06540-y

**Published:** 2021-06-07

**Authors:** Linda Takamine, Jane Forman, Laura J. Damschroder, Bradley Youles, Jeremy Sussman

**Affiliations:** 1 Center for Clinical Management Research, VA Ann Arbor Healthcare System, 2215 Fuller Rd, Ann Arbor, MI 48105 USA; 2 Department of Internal Medicine, University of Michigan, Ann Arbor, USA; 3 Institute for Healthcare Policy and Innovation, University of Michigan, Ann Arbor, USA

**Keywords:** Risk prediction, Cardiovascular disease prevention, Provider behavior, Implementation

## Abstract

**Background:**

Although risk prediction has become an integral part of clinical practice guidelines for cardiovascular disease (CVD) prevention, multiple studies have shown that patients’ risk still plays almost no role in clinical decision-making. Because little is known about why this is so, we sought to understand providers’ views on the opportunities, barriers, and facilitators of incorporating risk prediction to guide their use of cardiovascular preventive medicines.

**Methods:**

We conducted semi-structured interviews with primary care providers (*n* = 33) at VA facilities in the Midwest. Facilities were chosen using a maximum variation approach according to their geography, size, proportion of MD to non-MD providers, and percentage of full-time providers. Providers included MD/DO physicians, physician assistants, nurse practitioners, and clinical pharmacists. Providers were asked about their reaction to a hypothetical situation in which the VA would introduce a risk prediction-based approach to CVD treatment. We conducted matrix and content analysis to identify providers’ reactions to risk prediction, reasons for their reaction, and exemplar quotes.

**Results:**

Most providers were classified as Enthusiastic (*n* = 14) or Cautious Adopters (*n* = 15), with only a few Non-Adopters (*n* = 4). Providers described four key concerns toward adopting risk prediction. Their primary concern was that risk prediction is not always compatible with a “whole patient” approach to patient care. Other concerns included questions about the validity of the proposed risk prediction model, potential workflow burdens, and whether risk prediction adds value to existing clinical practice. Enthusiastic, Cautious, and Non-Adopters all expressed both doubts about and support for risk prediction categorizable in the above four key areas of concern.

**Conclusions:**

Providers were generally supportive of adopting risk prediction into CVD prevention, but many had misgivings, which included concerns about impact on workflow, validity of predictive models, the value of making this change, and possible negative effects on providers’ ability to address the whole patient. These concerns have likely contributed to the slow introduction of risk prediction into clinical practice. These concerns will need to be addressed for risk prediction, and other approaches relying on “big data” including machine learning and artificial intelligence, to have a meaningful role in clinical practice.

**Supplementary Information:**

The online version contains supplementary material available at 10.1186/s12913-021-06540-y.

## Background

Within the last 10 years, the clinical practice guidelines for blood pressure reduction, the use of aspirin, and the use of cholesterol-lowering statin drugs have given risk prediction a central role in clinical decision making [1–3]. In fact, in all major clinical practice guidelines, cardiovascular risk has a larger role in clinical decisions than it did 10 years ago [4–6]. This is due primarily to research showing that using risk prediction to guide these decisions, rather than traditional guidelines more strongly based on individual risk factors, could prevent more heart attacks and strokes while using fewer medications [7–9]. Since then, at least one large risk-driven intervention showed substantial clinical benefit [10].

The introduction of risk to CVD prevention is part of an ongoing shift in the paradigms of medical treatment. Recommendations for clinical decisions rely less on purely biological models that are dependent on blood pressure, cholesterol, and other biomarkers. New guidelines are using quantified decision models in which risk prediction, which relies on calculations from large datasets, is playing a growing role in many medical decisions [1, 11–19]. The importance of risk prediction in medicine, and the associated topics of Big Data and Artificial Intelligence, has grown by fits and starts, but the potential for benefit and public interest remains very large [10].

Despite this, multiple studies have shown that patients’ risk still plays almost no role in current clinical decision-making in CVD prevention [1–3, 14, 15, 20, 21]. This is not entirely surprising, since guidelines alone have repeatedly been shown to be inadequate in driving wide-spread implementation of new practices [1, 12, 20, 22]. Tools to help encourage the use of these guidelines, and to improve cardiovascular prevention in high-risk patients, have had an effect, but only a small one [23, 24].

Existing programs to adopt risk in clinical decisions vary by guideline-maker and country. Most major guidelines currently use a simple risk score that incorporates some combination of age, gender, cholesterol levels, blood pressure levels, smoking status, diabetes status, and at most 2–3 other variables. All common guidelines have nomograms available for in-clinic, technology-free use, but are also incorporated into handheld and online apps and are often calculated in electronic health records systems. One exception to this is the British guidelines, which recommend use of the QRISK3 tool [25]. QRISK3 does have an online tool, but it uses over 20 variables and is built to be automatically calculated within the local providers’ electronic health records. In the US, the United States Preventive Task Force recommends statin use for patients with “a calculated 10-year risk of a cardiovascular event of 10% or greater” for people ages 40–75 [26]. The American Heart Association recommends statins in patients with a 10-year risk > 7.5% in the context of a risk discussion [23]. Both groups recommend using the Pooled Cohort Equations [27]. The British National Institute of Clinical Excellence recommends statins and lower thresholds for hypertension control in people with a 10-year risk > 10%, as measured by QRISK3 [25, 28, 29], which is easily available to British medical providers. The European Society of Cardiology’s recommendations are a combination of risk prediction using SCORE [30] and individual risk factors [31].

In addition to this variation, individual risk models are controversial. SCORE has shown inconsistent validity in different European countries, which they have accounted for by creating local variations [32]. The Pooled Cohort Equations are criticized as predicting too many cardiovascular events and being inaccurate in Black Americans [33, 34]. The appropriateness of individual risk thresholds is inherently subjective and varies by guidelines.

What is unknown, though, is why providers are uncomfortable with using risk in their clinical decisions. Possible explanations are that using risk may be impractical, that they disagree with the guidelines, that they worry patients disagree with the guidelines, or many others. Therefore, we sought to understand providers’ views on the opportunities, barriers, and facilitators of incorporating risk prediction to guide their use of cardiovascular preventive medicines. We used a descriptive qualitative methodology grounded in a naturalist philosophy, wherein the goal is to be “data-near,” reporting findings in their everyday terms in order to more directly reflect participant experiences rather than higher-inference interpretations of the researchers. This approach supports our goal of producing concrete findings for real-world practice [35].

## Methods

### Sampling and recruitment

The Veterans Health Administration (VA) is the largest integrated health care system in the United States, providing care at 1255 health care facilities, including 170 medical centers and 1074 outpatient sites of care, serving 9 million enrolled Veterans each year. For administrative purposes, VA is divided into 18 regional Veterans Integrated Service Networks (VISNs).

We recruited VA primary care providers in the Veterans Integrated Service Network (VISN) region 10, which includes Michigan, Ohio, Northern Kentucky, and Indiana. Using a maximum variation approach, facilities were ranked according to their geography, size, proportion of MD to non-MD providers, and percentage of full-time providers. Upon ranking facilities, we sampled, on a rolling basis, a group of facilities that included variation on each of these characteristics. We contacted chiefs of staff and facility directors via email to elicit interest for participation of their facilities’ primary care providers in interviews. We gave facility leadership the option to opt-out of the project within 10 days or, if they were interested, we asked them to connect the research team with a point of contact at their primary care clinic. If facility leadership did not respond or opt-out within the 10-day period, the study team generated a contact list of providers from the VHA Support Service Center (VSSC) reporting system and recruited providers directly. Of 12 facilities contacted, 9 agreed to participate in the study (1 health system with 17 facilities had opted out at the health system level). At 7 of the 9 participating facilities, the clinic point of contact sent project and research team contact information to all eligible providers, who could contact the team to schedule an interview if interested. For the remaining 2 participating facilities, at one, the clinic point of contact provided the study team with a list of providers for the study team to contact directly and elicit interest for being interviewed; at the other, facility leadership did not respond within the 10-day opt-out period and study staff recruited providers directly via email. Finally, 2 providers contacted the study team after a colleague who had previously completed an interview referred them. Providers were defined as MD/DO physicians, nurse practitioners, physician assistants, clinical pharmacists, and nurse managers.

We selected primary care providers since they provide the large majority of primary preventative management in VA and elsewhere. We interviewed clinical pharmacists because they greatly influence CVD treatment. The project was approved by the Ann Arbor VA Healthcare System Institutional Review Board.

### Data collection

The study reported here was part of a larger study that examined two aims: 1) understanding primary care providers’ approaches to CVD prevention and comparing their approaches to clinical guidelines; and 2) characterizing providers’ reactions to moving to a risk-based approach to CVD prevention. This study focuses on the second aim. For this part of the study, we introduced a hypothetical situation in which performance measurement in the VA would be based on how much they reduced a patient’s 10-year CVD risk to a “realistic goal” as opposed to whether they met individual targets for blood pressure and cholesterol (a “treat to target” approach). We piloted the guide with three providers at VA Ann Arbor Health System, revising questions that failed to elicit intended responses or were difficult to understand. Two qualitative researchers (LT, a medical anthropologist and JF, a health services research qualitative methodologist) conducted the interviews in person (24) or by phone (9) at the provider’s place of work from June to November 2018. The interviews varied in length from approximately 30 to 60 min. Interviews were audio-recorded using a digital voice recorder and transcribed. We stopped recruiting providers when we had achieved “code saturation” for all reported themes, as well as “meaning saturation” for all reported themes except the first [36]. As the only conceptual (vs. concrete) theme, it is possible that additional interviews would have revealed additional dimensions of that theme. However, in our judgment as experienced qualitative researchers, data for all themes were adequately robust to support our findings and fulfill the aims of the study [30].

### Data analysis

LT and JF analyzed transcripts using directed qualitative content analysis [37, 38], with both deductive and inductive elements, and matrix analysis [39, 40]. Analysis was concurrent with data collection. First, they developed preliminary codes based on the interview guide, then refined these codes and added new ones that they identified through immersion in the data (interviewing and reading transcripts), in consultation with the wider study team (JS, a primary care physician and the principal investigator; LD, an implementation science expert; and BY, a research associate). Codes were used to create a structured summary template, which summarized each transcript in detail. Codes relevant to the present study were: providers’ reactions to moving away from a treat to target approach and toward the hypothetical shirt to risk prediction, reasons for providers’ reaction (identified inductively), and exemplar quotes. LT and JF summarized transcripts independently, then reconciled summaries based on discussion. After demonstrating that the analysts reliably extracted the same information from each transcript (after reconciling 13 transcripts), only one analyst (LT) completed them. Summary data by code and subcode relevant to this study were entered into a partially-ordered descriptive cross-case matrix that provided a comprehensive look at the data set [40, 41]. JF and LT then classified interviewees into 3 groups based on patterns in the data that indicated their reaction to moving away from the treat-to-target approach and toward the hypothetical shift to risk prediction. The 3 groups were then compared, and a comprehensive set of quotes were compiled by LT and JF for each theme, for each group, by manually reviewing both the transcript summaries and the original transcripts, and entering them into an Excel spreadsheet. Findings were developed with input from the wider research team through discussion in meetings and review of manuscript drafts.

## Results

**Fig. 1:**
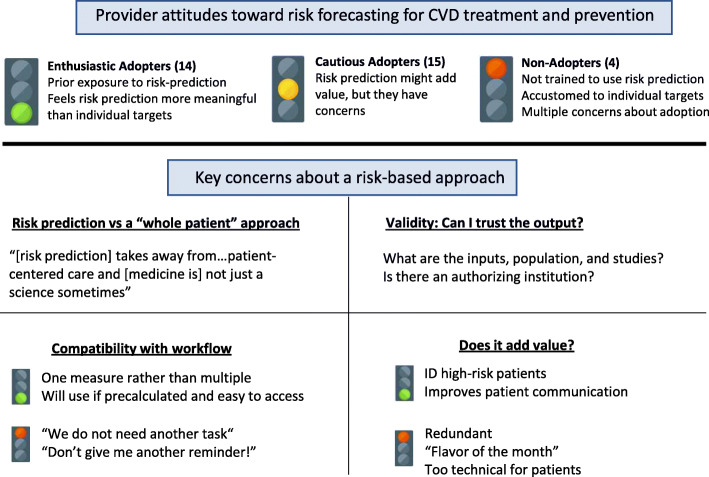
Interview results

We interviewed 33 providers: physicians (18), nurse practitioners (12), and clinical pharmacists (3) at 9 sites. While we attempted to recruit physician assistants, there were few, and none of them responded to invitations to be interviewed. We excluded the 2 nurse managers who participated in the larger study from the present analysis because they did not describe their individual responses to shifting to risk prediction. Our analysis focused on provider attitudes towards adopting risk prediction and their key concerns about adoption. See Fig. 1 below for a summary of findings.

### Attitudes toward adoption of an ASCVD risk prediction-based approach

We identified three groups of providers: Enthusiastic Adopters (EA; *n* = 14); Cautious Adopters (CA; *n* = 15), who wanted key concerns to be addressed before adoption; and Non-Adopters (NA; *n* = 4), who rejected the approach. Supporting quotes for each group may be found in Table 1.

Enthusiastic Adopters often had prior exposure to risk prediction, mostly through training, and were already using risk calculators. One PCP said:

I can’t even imagine why [providers] wouldn’t [adopt risk prediction] because it’s been so thoroughly investigated and studied…[I use a risk calculator] every single day on every single patient…Even when I have them on statin, when I have them come back I look at their blood pressure, we run the numbers again. (10002EA)

All three of the clinical pharmacists we interviewed were Enthusiastic Adopters. One elaborated upon the role of training:

When the new cholesterol guidelines came out in 2013, that’s when…I changed my approach…in school we are taught guidelines, we are taught evidence-based medicine, we are taught comorbidities, we are taught medication focus...whereas I feel like providers are taught patho-phys and these are two drugs that might work. (10020EA)

Another clinical pharmacist felt that the goal of reducing CVD risk would be more meaningful than performance measures based on treat to target because it considers morbidity and mortality:

I think it would be a little bit more meaningful and it also would be more applicable to what research is currently looking at, given that so many of the trials now are more looking at for medications are they cardiovascular protected, do they decrease morbidity and mortality and hospitalizations versus how much they lower blood pressure or how much they lower your cholesterol. (10018EA)

Cautious Adopters described being more comfortable with treat to target, or had other concerns, which we describe in the next section:

“it’s definitely the better way to go but it’s, I guess it could be easier I suppose, I don't know, but when you’ve been practicing so long and looking at simple target proxy improvements, that’s what you get used to.” (10003CA)

Non-Adopters were unfamiliar with the approach, had many concerns, and saw little value in it. One provider, when asked whether they would want the VA to adopt a risk prediction approach, said:

"Probably not…and that’s probably because, with the medical training, they’re teaching us to focus on the hypertension and the high cholesterol and the weight and the this and that." (10001NA)

**Table 1:** Provider stances toward adopting risk prediction for CVD treatment and prevention

Enthusiastic adopters	“I would be the happiest man in the world because right now today my nurse did a printout of all my patients with high blood pressure that are above the 140/90, and so we’re going to, she’s going to try to call them and see if we can help them and find out what’s going on, and we wouldn’t be doing that, we’d be focusing on patients as we see them and going from there. So we’re doing a lot of wasted, in my humble opinion, we’re chasing numbers, we’re not chasing patients and that doesn’t do anybody any good except it makes the VA look good on Fox news or wherever, whoever’s counting the beans and wants to say, ‘See? We’ve got 95% of the VA patients here are at goal!’ It doesn’t matter they’re all dying, but they’re at goal and it’s irrelevant” (10028EA) (PCP, physician)
Cautious Adopters	“I would be a little uncomfortable with [abandoning treat to target] because we’re based on targets that they’re practiced, but you know I’m willing to abandon it if I can get comfortable with whatever goal we go with.” (10003CA) “I would take whatever advice you give me and I think it would help me in my thinking though. It doesn’t mean that I have to stick to it…, but at least it will give me some guidance. I think it should still be there, I don’t think it is authoritative.” (10011CA)
Non-adopters	“I guess I’m just used to the guidelines and we just do them” (10001NA) “I’m not going to ignore other targets which I traditionally follow…if they give me a list of my patients who are not under control, I do pay attention to that.” (10031NA)

### Key provider concerns

Providers’ key concerns fell into four categories: 1) potential conflict between quantified risk goals and a “whole patient” approach; 2) uncertain validity of the risk prediction model; 3) potential workflow burdens; and 4) the value of introducing risk prediction. Supporting quotes may be found in Table 2 at the end of the results section.

### Quantified risk goals vs a “whole patient” approach

Most providers across all three groups referred to bringing a “whole patient” approach to their treatment decisions. To these providers, whole patient care includes consideration of lifestyle behaviors; comorbidities; physician-patient communication and trust; quality of life issues such as stress, anxiety, and depression; socioeconomic status and its effect on adherence and access to care; and finally, medication issues such as interactions and side effects. Many worried that a risk prediction approach would preclude consideration of these difficult-to-quantify factors.

Non-Adopters expressed the most concern about omitting whole-patient factors. They felt that risk prediction was overly reductionist and that it represented an inadequate, cookie-cutter approach to patients:

There’s so many different variables and [quantification] takes away from…patient-centered care and it’s not just a science sometimes. (10001NA)

What’s [the patient’s] socioeconomic status? What’s his other factors? Can he afford more things that we are recommending? Is he having problems with those medications that he’s comfortable communicating? Every person is so individualistic that you can’t treat a person just on the basis of numbers…for example we say “You should just do fruits and vegetables and all these healthy things,” and they might not tell you that they can't afford to buy it and it’s only if you develop that patient trust that you can find out…all he can afford is buying snacks and chips and crackers…You have to treat the person as a whole... (10009NA)

In contrast, both enthusiastic and cautious adopters felt that risk prediction can fit into a whole person approach:

I think [adopting risk prediction] is a great idea…the goal is to get everyone under control with everything, but...we treat the patient, not the numbers anyway, so it’s a guideline for us to reach that goal...I see the whole patient... (10027CA)

One clinical pharmacist took the stronger view that risk prediction facilitates a whole person approach compared to treat to target:

…their blood pressure is a little bit high but…do I care that his blood pressure is high? If he's falling all the time I can't push him too low. So if I have a risk stratification moving forward, it helps me define my target of therapy a little bit better regarding medications, versus, I think it's treat to target…with heart failure and cardiovascular disease being on certain medication classes decreases your risk versus what the blood pressure actually says and if you can tolerate it. So I think if you go to a risk stratification that’s going to help you treat the patient as a whole versus treating the numbers. (10020EA)

### Validity of risk prediction

Providers in all three groups wanted to assess the validity of the risk prediction model being used, including how patient risk was calculated, factors included in the risk equation, and supporting evidence:

Who’s determining the realistic goal and what would make me trust it? That is tricky, I mean for me, it would have to go back to evidence-based medicine. So if there were these composite studies, these cohorts that came out and said this is how we calculate our realistic goal, this is how effective it was, these are the heart attacks we prevented or something…it goes back to studies. (10020EA)

One provider was concerned about whether a professional institution such as the American Heart Association (or American Academy of Family Physicians) had endorsed the risk prediction model:

What we do know is that we’re supposed to listen to our Academy of Family Practice or Diabetic Association or whatever else…the more those types of organizations that recognize that, “Hey, this is a good way to evaluate your patient panel,” I think you know the more confident I am in being able to follow that. (10035CA)

### Compatibility with workflow

Given the time pressures they face, concerns about workflow were common in all three provider groups. Concerns included calculating or searching for risk numbers for each patient:

You’re creating, going to create a reminder or something in there, what else are you going to take off my plate? …they have a way of adding things but not necessarily removing something else… (10031NA)

…if there’s something that populates that information right away, I think it would be helpful… you’re pushed for time, there’s so many other things we need to look at (10033EA, 320–5)

A few also mentioned the cognitive burdens associated with switching to this novel method:

I think it’s easier to treat a blood pressure down to a certain value; it’s easier to treat an A1C down to a certain value than, “Well, if this person’s A1C is 7.5, the cardiovascular risk is a certain number, and if we get it down to 6.8, the cardiovascular risk is a different number,” whereas just going from 7.5 to 6.8 it just seems easier to comprehend and to work on (10032CA)

### Does adopting risk prediction add value?

Providers in all three groups considered whether adopting risk prediction would actually add value to their day-to-day practice. They saw value primarily in risk stratification and patient communication. Critics felt that risk prediction was redundant to existing procedures, appeared to be another fad, and would confuse patients.

Some providers said that risk prediction could clarify risk for patients who “look good on paper”; e.g., patients with diabetes whose blood pressure goal should be lower, or patients with LDL and blood pressure below target levels but who have other risk factors such as age. Others mentioned that risk prediction could decrease cognitive burden in prioritizing patients:

…it's right there in a nice picture, easy visual form…if you have a patient case it's hard to say , “Okay, this one would benefit the most and this one,” plus it’d be time-consuming to read through all of them whereas [with pre-calculated risk figures] it’s like, “Okay, that’s the one we’ll start with…” (10016EA)

Many Enthusiastic and Cautious Adopters felt that risk prediction would improve communication and bolster shared decision-making by enhancing patient understanding:

So instead of telling my patient, “Look, you know I need to get your blood pressure below this because it reduces your cardiovascular risk,” I've got actual numbers to show them. So, “Look, right now your risk is 30% over 10 years that something is going to happen, but if we give you these medications, I can reduce that to 25%.” …I think in most cases seeing those kinds of numbers are relatable to people. (10036EA)

Some thought that giving patients a “visual” of their potential risk reduction would motivate them to adhere to treatment:

I think it’d be motivating to them because they'd be able to see the, so if you put it in just as an example, “If we get your cholesterol down below 100 it decreases your risk of having a heart attack by 10%,” 10% is pretty significant, for most people that would be important to them (10024CA)

Regarding doubts about added value, several Cautious and Non-Adopters felt that risk prediction was redundant because they already perform similar calculations mentally. As one clinician said, “It’s not like we don’t calculate the risk, but you’re trying to treat the person as a whole” (10009NA). Given the number of quality improvement initiatives at the VA, risk prediction seemed to some like “just a different spin on what we currently have” (10019CA). Some felt risk prediction may be too technical for patients, who are used to talking about individual cholesterol and blood pressure targets.

### The role of performance measurement

Nearly all providers said that performance measures play no role in their treatment decisions, and that they comfortably disregard performance measures in favor of what they feel is best for the patient. Most providers did not feel that performance measures reflect quality patient care. A number objected to being evaluated on the basis of unmodifiable risk factors, and speculated that performance measures based on risk prediction could have negative unintended consequences for patients (see Table 2).

**Table 2:** Key concerns about adopting a risk prediction-based approach to CVD treatment and prevention

Key Concerns	Representative Quote
Quantified medicine vs Whole person approach	“a person is a person, you cannot formulize and say, one and one is two… one plus one doesn’t always add up to two. There’s so many other factors that are there that changes the way you think…so the comorbidities and…medications that will interact with it, all these things has to be factored in in order to see whether you can really get to that goal or not.” (10011CA) “Motivation levels, access to care, ability to meet dietary goals based on a lot of different factors that play into a person making the necessary changes to be healthy. And when you say realistic goals, well, you can tell a person to not eat fast food, but if that’s what they can afford, they won’t change. (10023EA) “…medication burden and how that negatively influences adherence I think is something to take in…like adherence vs. medication burden and if it’s worth a whole other agent to get like 1% reduction. Um, and then also like fall risk, risk of adverse reactions for them.” (10016EA – clinical pharmacist)
Validity of Risk Model	“I mean what does this really mean, so break it down...show me the studies.” (10021EA, 299–302) “If somebody says, ‘Do you want to get it down to [the reasonable goal],’ I would say, ‘I need to know how you calculated It.’” (10029EA) “Which population did you do in your study, you know what focus was studied, are they applicable to the other populations?” (10031NA)
Impact on Workflow	“…perhaps if there was, like in a little box, you know like on the kind of the main page of the patient where it would say you know, what these numbers are so that it could be something we could reference without having to “answer to” such and such a reminder” (10036EA) “No, it needs to be maybe a little side note somewhere, even in their office or somewhere that the providers can look at, but we do not need another task (*taps table with each syllable for emphasis*). We can’t, no.” (10001NA) “I have to plug the numbers in, okay yeah, so I’ve got another 10 min to sit in front of you and say, ‘Let me see, oh your blood pressure is this, oh, that’s a + 2. Let me see what your cholesterol, oh, + 5. Oh, here’s your magic number, oh, now your risk factor is 12.8%, how can I make that better?’ I had to do all the work, well I’ve already got a lot of work to do. Now if you could do the work for me and you’re going to say here’s your magic numbers, Dr. X., I’m all right with that.” (10028EA)
Does risk prediction add value?	Adds value: *By Identifying High-risk Patients* “I wouldn’t have the same like blood pressure goals for everybody, so the hard part is some people that let’s say they have diabetes and micro albuminuria or macro albuminuria, they really should be less than 130/80 for their blood pressure, but if you look in like I think it’s our SAL or our HEDIS or whatever it is, when you pull it up, those patients wouldn’t be identified and they really should be on… an ace inhibitor for their risk but it doesn’t necessarily show that, so it’s like those people kind of get lost in the shuffle…So I think it’d be better to try to move to you know a system that shows like 10 year risk and who to target the most.” (10016EA) *By Improving Communication with Patients* “I would use it as a tool to show the patient that didn’t want to take medication…and say, “Well, you’ve got a 30% chance now, a 10-year chance of a heart attack or stroke with no medication,”…that might help to sway them to take medication.” (10000CA) “… it’s a visual for them and I think visuals really tend to you know, be a good thing in practice…if they see numbers, maybe cut in half that’s pretty impressive, even with just you know smoking cessation, how significantly the numbers drop or even weight loss, 5% you know with a reduction in your blood pressure…I think it gives them incentive you know to strive to be healthier.” (10002EA) “‘Your risk is 13% without no medication, if we put you on, optimize your medications, we can get your risk down to 6%,’ I mean most people even if they don’t have a good health literacy can understand that 6 is less than 13” (10015EA) May not add value: “…you could say that everybody has to be within 2% of the realistic goal, and have 30 people that are 2.1% [within] the realistic goal. Is it worth bringing them in, beating them over the head and say, ‘I’m going to give you a fourth blood pressure medicine because if I just got back down, my numbers would now be at two and I will get bonus, but if not, my poor grandkids won’t go to Disneyland,’ so we’re still playing that silly little game of numbers. But I think it’d be easier rather than doing different areas and trying to chase your tail all the time. You know this week it’s hypertension, next week I’ll get yelled at for diabetics, the weekend after that it’s cholesterol, the weekend after that it’s you doing up mammograms I mean it just we’re always chasing your…If all my patients are 0.2 away from their realistic goal, are 35 years old, it would be more concerning. If all of my patients that were 2.2 away from their goal are 85, those are two different people, two different populations at two different levels in their life to be worried about 0.2.” (10028EA) “Yeah, so some risks are modifiable and some are not, like if there is family with hyperlipidemia, you can’t, but if you modify the hyperlipidemia and get to at goal, this may not be at goal for a person oh you know who has a family history of premature coronary artery disease, you want it lower the better, things like that. So you go from the person’s risk, history and based on the smoking, smoke cessation is what you can alter, blood pressure number is what you can alter, which is what we are talking about…so that kind of stuff, and weight.” (10012CA) “It’s not like I’m saying “No you can’t do this,” but will this current goal pressure on the physician help that? I don’t think so.” (10012CA)

## Discussion

The principal objective of this study was to explore providers’ views on adopting a risk prediction-based approach to CVD prevention. Generally, providers were concerned that risk prediction can be reductionist, with some worrying that it would replace their judgment and their whole patient approach with algorithms.

We hypothesize that this central conflict will also occur in other examples of risk prediction in clinical care. Despite major public interest, the adoption of big data, risk prediction, and artificial intelligence in medical care has generally been slower than in other fields [42]. Other research has found that hospitals and health system leaders have had concerns similar to those we found for providers [43, 44]. Concerns about conflicts with a whole person approach will be especially likely since treatment by risk prediction generally replaces the more biologically-driven approaches aligned with providers’ training and practice. Their worries about algorithms replacing their judgment are based upon skepticism about what algorithms can realistically predict, particularly with regard to patient lifestyle behaviors and social determinants of health.

Our findings are consistent with research on adoption of new practices more broadly. Decades of implementation science research shows that successful adoption requires many accompanying changes, often including technology, peer support, and changing organizational climate [45]. Work in cardiovascular risk prediction has shown concern that the specific needs of the healthcare system, users, and specific tools for adoption are often not met [45, 46]. This study differs from most previous research in that it applies that research to adoption not of a new drug or guideline, but to paradigm shifts in how providers think about treatment. Adoption of risk prediction has been slow because it has often not been accompanied by changes to address the key concerns that we found.

Addressing the key concerns we encountered will not always be straightforward, but we do believe it will be central to the effective use of technology in medicine. Providers’ concerns about validity of a risk prediction tool show that support from a trusted organization will be necessary though not sufficient. Consistent evaluation of the impact and effectiveness of both the guidelines and the underlying risk scores will be necessary. Compatibility with workflow could be partially addressed through technological innovation and practice reorganization. Not doing so will likely prevent adoption entirely. To convince providers that the tool will add value to clinical practice, providers must understand benefits such as improved outcomes and better patient communication. Incorporating risk prediction into a whole person approach to care will probably be the most difficult. Many providers did not understand how the two could be aligned. However, many providers saw how a risk prediction tool could possibly strengthen their whole person approach to care. Involving clinical pharmacists, who in this study showed a sophisticated understanding of the approach, may be fruitful.

Performance measurement and accompanying pay-for-performance systems have been seen as promising tools to improve healthcare implementation. Our findings recommend caution about using this approach. The providers we interviewed not only had largely negative views about performance measurements, they said such measures barely impacted their clinical actions at all, and also worried that improperly structured performance measures may reduce provider motivation and negatively impact patient care [47, 48].

This study is one of the most thorough examinations of the adoption of risk prediction in clinical practice to date. It is limited by whether providers’ insights about their own care are accurate, and the possibility that our providers are atypical for this problem, both due to the sample size and being VA providers. Concerning the first theme reported in Results, while we reached “code saturation” and report rich findings that are supported by our data, limited study resources for additional interviews leaves it unclear whether we reached “meaning saturation”; it is possible that additional interviews would have revealed additional dimensions of that theme [36].

## Conclusion

In general, we found that primary care providers accepted the potential value of risk prediction in CVD prevention, but many had misgivings, which included concerns about impact on workflow, validity of predictive models, the value of making this change, and possible negative effects on providers’ ability to address the whole patient. These concerns have likely contributed to the slow introduction of risk prediction into clinical practice. These concerns will need to be addressed for risk prediction, and other approaches relying on “big data” including machine learning and artificial intelligence, to have a meaningful role in clinical practice.

## Supplementary Information

**Additional file 1.**

## Data Availability

The datasets generated and/or analyzed during the current study are not available due to participant privacy but may be available from the corresponding author on reasonable request.

## Abbreviations

CA: Cautious Adopters
CVD: Cardiovascular disease
DO: Doctor of Osteopathic Medicine
EA: Enthusiastic Adopters
MD: Doctor of Medicine
NA: Non-Adopters
PCP: Primary Care Physician
VA: Department of Veterans Affairs (United States)
VISN: Veterans Integrated Service Network
