# Supplementary material for: Understanding providers’ attitudes and key concerns toward incorporating CVD risk prediction into clinical practice: a qualitative study
Source: BMC Health Serv Res. 2021 Jun 7;21:561. doi: 10.1186/s12913-021-06540-y (PMC8185928; doi:10.1186/s12913-021-06540-y)
Supplement: Supplementary file 1 — Additional file 1. [file 12913_2021_6540_MOESM1_ESM.docx]

**Provider Interview Guide**

**Introduce self and purpose of interview**

Hi, I’m ____________. Thank you for taking time out of your busy schedule to speak with us. I’ll give a quick reminder about why we’re here. We are not clinicians—we’re qualitative researchers and this is a research project about how providers make decisions about cardiovascular care, and how the VA might affect those decisions. We are interviewing people across several VA facilities; we’re not here to evaluate you, we’re just interested in the variation in how provides approach cardiovascular disease.

The interview should last under an hour.

**Confidentiality**

**[Hand information sheet to interviewee]**

I’m going to take a little time to read to you our efforts to protect confidentiality.

The interview will be completely confidential. We plan to record it, but you can ask that the voice recorder be turned off, decline to answer any interview questions, or stop the interview at any time.

- The recording will be uploaded and deleted from the recording device today.
- A project transcriptionist will transcribe the interview.
- Any written notes taken by the team will be stored in a locked file cabinet.
- All audio, notes, and transcripts are stored behind the VA firewall in a folder accessible only by specific project staff.
- If we ever share anything we learn during these interviews with people outside of our project team, we will de-identify everything you said and it will not be traceable to you.

Do you have any questions for me before we begin?

I’m turning on the recorder now.

**Section 1: Background Information**

I’d like to start off with some background questions to learn about you as a clinician. What are your main professional roles and responsibilities at the VA?

- How long have you been in this role?
- Are you full/part time?
- What size is your panel? Is it relatively large/small?

**Section 2: Patient scenarios**

Thank you. From this point onwards, the interview will be in two parts. The first part will involve four patient scenarios on these cards. The second part will involve another visual aid which I’ll give you at that time.

Each of these cards has the patient’s name and a description of the patient’s clinical situation. Again, there is no right or wrong answer; we’re interested in how providers differ in their approaches to cardiovascular disease, and we want to understand that variation.

If you could please first read through these scenarios and just familiarize yourself with them. Then I’ll ask you a few questions about your treatment approach.

[**Give cards to provider. Provider reads scenarios]**

Thank you. Now, if you could place the cards in rank order based on how important cardiovascular preventative medications are, starting with the most important and on down the line, and think aloud while you do it so we can understand your thought process.

Now could you think aloud as you formulate a treatment plan aimed at CVD for each.

***Probes:***

**[If they ask about any additional factors that are not in the cases]**

We’d like you to focus on the information on the cards, but please tell us what factors might change the actions you take with this patient?

**[If they mention additional factors to consider]**

You mentioned many factors that affect your approach to treatment: What factors do you prioritize? Say, the top three

**[If they mention lifestyle changes]**

How do you balance the timing and priority of lifestyle changes and prescribing medication?

**[If they mention risk tool]**

Clarify what risk tool they use

- What **resources** do you primarily draw upon when thinking about treatment priorities; for example, guidelines, talking to other providers, journal articles or lay press, internal educational programs, clinical experience.
- **[Only if NOT evident from scenarios]** While you were formulating treatment for each patient, were your choices guided by some vision of what **“good quality care”** is?
- What do you think **“poor quality care”** is? [If they emphasize lifestyle/behavioral changes: How do you balance the use of medications with efforts on the part of the patient to alter their lifestyle?]
- **[If NOT already answered]** How do you incorporate **patient preferences** and communicate treatment options?

***Performance measures***

- If and when they think about **PMs/evaluations** of how they approach CVD, whether during patient visit or at some other time
- Do you feel PMs reflect whether you deliver good or poor quality care?
- Have you experienced conflicts between PMs and what you consider good quality care? If so, how did you handle that conflict?

***Audit and Feedback***

- Based on current PMs, all of the patients require further action, and they would appear on a feedback report if you did not take action. How do you feel about this?

***General A&F questions***

- Do you receive reports about the quality of your care from your site? If not, would you like it?

(description—patients who not meetings standards; how well providers are meeting quality measures, sometimes with examples of patients who are not meeting

- Do feedback reports help you understand how you might improve your performance?
- Do you feel feedback reports help you understand which patients should be higher priority for follow-up? How could they best indicate that, or do you have thoughts on how they are unhelpful in this regard?
- Are there repercussions for not following up?

***Exception reporting***

- Do you feel that any of the patients should be excluded when your performance is being evaluated? If so, why; if not, why not.

***If there is time:***

- **What could the VA do** to help you provide what you consider good quality care?
- **What could the VA do** to demonstrate an understanding that you are attempting to provide good care?
- Is there anything that pushes you away from the care you think you should be providing?

**Section 3: BTT**

Now we’ll get into the second part of the interview, which involves a new, hypothetical approach to treating cardiovascular disease. Let’s suppose that the VA has announced that it will move completely away from using specific targets to assess performance, such as getting BP below 140 or 130. Instead, you will be given information on patient’s 10-year risk of developing cardiovascular disease. I’m going to give you a couple of visual aids that contain that risk information for each of the previous patient scenarios. **[Hand visual aids to interviewee. Explain table and chart to interviewee]**

- If you had this additional information, would it **affect your earlier treatment decisions** in the scenarios? For example, are there any medicines or studies might you use differently?
- How would you feel about hypothetically **abandoning a focus on individual targets**?
- If they say they already incorporate risk, ask them about how they came to use that approach
- What kind of an impact do you think this new approach would have on patient care? What about communication with patients?
- Probe about the **“reasonable goal” number**

***Barriers and facilitators***

- Clinical practice guidelines [from professional associations e.g., AHA] have incorporated risk for some time, yet it is not being used in practice. Why do you suppose this might be the case?

**Section 4: Clean-up questions [only ask if not addressed above and if there is time]**

***Audit and feedback:***

- If you had complete control, how would you like to receive feedback about your clinical performance?
- How would the feedback tell you whether you were doing a good job?
- What sort of benchmark would it be helpful to compare your performance against?
- Can you think of any helpful performance reports you’ve received? What made them helpful? What about unhelpful performance reports?

**PATIENT SCENARIOS**

**Patient 1**: Mr. Jones

Sex: Male

Age: 55

BP: 142/91 mm/Hg.

LDL: 125

HDL: 35

History: History of hypertension (previous blood pressure readings commonly as high as 180/90 mm/Hg)

Medication: started blood pressure therapy a year ago and is now taking 3 different blood pressure medications and 40 mg of atorvastatin.

**Patient 2**: Mr. Smith

Sex: Male

Age: 75

BP: 142/91 mm/Hg

LDL: 125

HDL: 35

History: COPD

Falling more frequently lately

Medication: 40 mg of atorvastatin

**Patient 3**: Ms. Clark

Sex: Female

Age: 38

BP: 142/91

LDL: 125

HDL: 35

History: Elevated blood pressure readings for the past two years.

Altered dietary/exercise regimen to follow recommendations.

Not interested in starting any blood pressure medicines.

Medication: None

**Patient 4**: Ms. Baker

Sex: Female

Age: 65

BP: 160/95 mm/Hg

LDL: 165

HDL: 28

History: Smoked half a pack of cigarettes (10 cigarettes) per day for the past ten years.

Medication: None

**Visual Aids**

**10-Year Chance of Heart Attack or Stroke**

|  | No medication | Current medication in scenarios | Realistic goal | Difference between current medication and realistic goal |
| --- | --- | --- | --- | --- |
| 1. Mr. Jones  55 YO man | 13% | 8.6% | 6.4% | -2.2% |
| 2. Mr. Smith  75 YO man | 30% | 28.5% | 25.3% | -3.2% |
| 3. Ms. Clarke  38 YO woman | 1.7% | 1.7% | 1.6% | -0.1% |
| 4. Ms. Baker  65 YO woman | 20.4% | 20.4% | 7.8% | -12.6% |
